# Beyond oil degradation: enzymatic potential of *Alcanivorax* to degrade natural and synthetic polyesters

**DOI:** 10.1111/1462-2920.14947

**Published:** 2020-02-27

**Authors:** Vinko Zadjelovic, Audam Chhun, Mussa Quareshy, Eleonora Silvano, Juan R. Hernandez‐Fernaud, María M. Aguilo‐Ferretjans, Rafael Bosch, Cristina Dorador, Matthew I. Gibson, Joseph A. Christie‐Oleza

**Affiliations:** ^1^ School of Life Sciences University of Warwick Warwick UK; ^2^ Unidad de investigación‐HUC La Laguna‐Tenerife Spain; ^3^ Department of Biology University of the Balearic Islands Spain; ^4^ IMEDEA (CSIC‐UIB) Esporles Spain; ^5^ Laboratorio de Complejidad Microbiana y Ecología Funcional Universidad de Antofagasta Antofagasta Chile; ^6^ Departamento de Biotecnología Universidad de Antofagasta Antofagasta Chile; ^7^ Centre for Biotechnology & Bioengineering (CeBiB) Santiago Chile; ^8^ Department of Chemistry University of Warwick Warwick UK; ^9^ Warwick Medical School University of Warwick Warwick UK

## Abstract

Pristine marine environments are highly oligotrophic ecosystems populated by well‐established specialized microbial communities. Nevertheless, during oil spills, low‐abundant hydrocarbonoclastic bacteria bloom and rapidly prevail over the marine microbiota. The genus *Alcanivorax* is one of the most abundant and well‐studied organisms for oil degradation. While highly successful under polluted conditions due to its specialized oil‐degrading metabolism, it is unknown how they persist in these environments during pristine conditions. Here, we show that part of the *Alcanivorax* genus, as well as oils, has an enormous potential for biodegrading aliphatic polyesters thanks to a unique and abundantly secreted alpha/beta hydrolase. The heterologous overexpression of this esterase proved a remarkable ability to hydrolyse both natural and synthetic polyesters. Our findings contribute to (i) better understand the ecology of *Alcanivorax* in its natural environment, where natural polyesters such as polyhydroxyalkanoates (PHA) are produced by a large fraction of the community and, hence, an accessible source of carbon and energy used by the organism in order to persist, (ii) highlight the potential of *Alcanivorax* to clear marine environments from polyester materials of anthropogenic origin as well as oils, and (iii) the discovery of a new versatile esterase with a high biotechnological potential.

## Introduction

In microbial ecology, the idea that ‘*everything is everywhere*, *but*, *the environment selects*’ (Baas‐Becking, 1934; De Wit and Bouvier, 2006) requires that even the rare biota has to persist through time via the assimilation of a source of carbon and energy, unless they are able to remain dormant such as by sporulation. Marine ecosystems are mostly oligotrophic and hostile to non‐adapted microorganisms due to the lack of nutrients and high salinity. The predominant heterotrophic bacteria that inhabit these environments are thus well adapted to such conditions and have mainly specialized in the use of labile substrates released by marine phototrophs (Sharma *et al*., 2014; Christie‐Oleza *et al*., 2017; Bakenhus *et al*., 2018; Zheng *et al*., 2019).

*Alcanivorax* is a ubiquitous marine bacterial genus classed as an obligate hydrocarbonoclastic bacterium (OHCB) due to its preference to metabolize hydrocarbons and crude oil derivatives (Yakimov *et al*., 2019). This genus rapidly blooms and becomes one of the most abundant organisms during marine pollution events and oil‐spills (Kasai *et al*., 2002; Hara *et al*., 2003); however, how does *Alcanivorax* persist within the rare biome of pristine seawater while awaiting favourable conditions? Although members of the *Alcanivorax* genus can grow – although inefficiently – with more labile substrates such as pyruvate and succinate (Fernández‐Martínez *et al*., 2003; Naether *et al*., 2013; Radwan *et al*., 2019), they would be outcompeted by other heterotrophic bacteria in the natural environment (McGenity *et al*., 2012; Yakimov *et al*., 2019). It has been suggested, although, that *Alcanivorax* spp. may persist in pristine environments by using alkanes released by marine cyanobacteria (Coates *et al*., 2014; Lea‐Smith *et al*., 2015) and other hydrocarbon‐producing eukaryotic algae (Sorigué *et al*., 2016, 2017). The hydrolysis of aliphatic polyesters such as poly(caprolactone) (PCL), poly(hydroxybutylate) (PHB) and poly(butylene succinate) (PBS) by some *Alcanivorax* isolates has also been shown (Sekiguchi *et al*., 2011; Zadjelovic *et al*., 2019), although the underlying molecular mechanisms behind the ability of these strains to degrade such polyesters remained unknown. Interestingly, during the screening for polylactic acid (PLA) esterases, researchers identified that the enzyme ABO2449 encoded by *Alcanivorax borkumensis* had a strong hydrolytic activity on PLA as well as on a range of other aliphatic polyesters, that is, poly(hydroxybutylate‐*co*‐valerate) (PHBV), PCL and poly(ethylene succinate) (PES) (Hajighasemi *et al*., 2016).

A large number of environmental microbes – including members from the genus *Alcanivorax* – synthesize intracellular aliphatic polyester granules, that is, polyhydroxy alkanoates (PHA), as a strategy to store carbon and energy when nutrient availability is imbalanced (*e*.*g*. under high C:N condition*s*; Fernández‐Martínez *et al*., 2003; Sabirova *et al*., 2006; Jendrossek and Pfeiffer, 2014). As well as aliphatic polyesters of natural origin (*e*.*g*. the PHAs: PHB and PHBV), in recent years, there has been an increase of industrially manufactured synthetic polyesters (*e*.*g*. PLA, PCL, PES or PBS; Flieger *et al*., 2003; Tseng *et al*., 2007). Aliphatic polyesters are tagged as ‘biodegradable plastics,’ and although they still represent a small fraction of the global polymer material market, the consumer demand of these environmentally friendly alternatives to traditional non‐biodegradable materials is exponentially growing (Tokiwa *et al*., 2009; Aeschelmann and Carus, 2015). Like all plastics, aliphatic polyesters also find their way into the oceans (Jambeck *et al*., 2015; Lebreton *et al*., 2017). Although considered biodegradable because their linking ester bonds are susceptible to hydrolysis by esterases, lipases or other enzymes (Nakajima‐Kambe *et al*., 1999; Müller *et al*., 2001; Zheng *et al*., 2005; Shah *et al*., 2014), other factors such as chemical structure, molecular weight, hydrophobicity and crystallinity may hinder degradability of these materials (Tokiwa *et al*., 2009). Furthermore, polymer degradation in marine environments is even more challenging due to the low temperatures and oligotrophic conditions that hamper microbial activity as well as the reduced encounter rate in such dilute ecosystems. Hence, although some marine microbial isolates have been reported to degrade a range of polyesters (Mabrouk and Sabry, 2001; Ghanem *et al*., 2005; Sekiguchi *et al*., 2010), such a process is not as obvious in marine environments as highlighted by studies that failed to observe degradation of some theoretically biodegradable polymers such as PHB, PES and PBS (Sekiguchi *et al*., 2010; Bagheri *et al*., 2017).

In this study, we characterize a novel and versatile esterase secreted by *Alcanivorax* that hydrolyses a number of natural and synthetic polyesters, that is, PHB, PHBV, PES, PBS and PCL. Altogether, the evidence here suggests that the environmental relevance of the genus *Alcanivorax* goes beyond the degradation of solely oil‐derived compounds.

## Results

### *Polyester degradation by* Alcanivorax *sp. 24*

**Figure 1:**
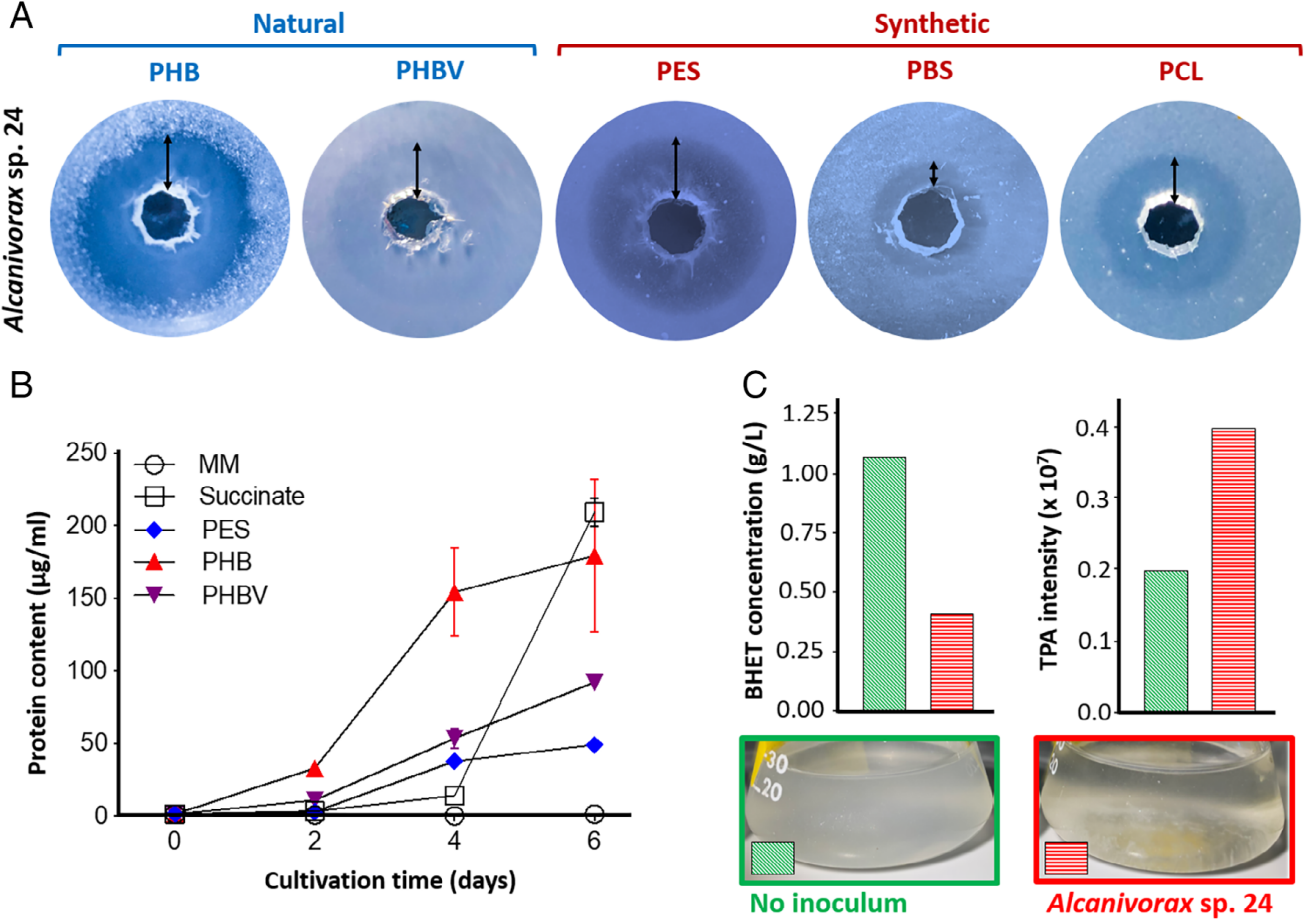
Determination of polyester degradation by *Alcanivorax* sp. 24.**A.** Clear zone hydrolysis test of *Alcanivorax* sp. 24 on the five aliphatic polyesters PHB, PHBV, PES, PBS and PCL. Polyesters were added at 0.3% w/v to BH mineral media containing 1% agarose (w/v). Arrows highlight the hydrolysis halos surrounding the 5 mm‐diameter wells made for *Alcanivorax* sp. 24 inoculation.**B.** Growth curves of *Alcanivorax* sp. 24 when incubated in the presence of three different polyesters, succinate (labile substrate control) and BH mineral media (MM; negative control). Increase in biological biomass was assessed by protein quantification. Error bars indicate the standard deviation of three biological replicates.**C.** Degradation of the PET intermediate BHET by *Alcanivorax* sp. 24. The substrate BHET and its hydrolysed product TPA were monitored by LC–MS. ‘No inoculum’ represents the replicate control condition where *Alcanivorax* sp. 24 was not inoculated. [Color figure can be viewed at http://wileyonlinelibrary.com]

*Alcanivorax* sp. 24, isolated from marine plastic debris (Zadjelovic *et al*., 2019), produced clear degradation halos on plates containing the polyesters PHB, PHBV, PES, PBS and PCL (Fig. 1A and Table 1). Although the degradation of the natural polymers PHB and PHBV was expected because the strain was originally enriched and isolated on PHB (Zadjelovic *et al*., 2019), the large degradation halos on the synthetic polyesters PES, PBS and PCL within just 3–6 days was remarkable and deserved further investigation. Growth curves of *Alcanivorax* sp. 24 were conducted with polyesters PHB, PHBV and PES to assess if the bacterium could assimilate and grow on these polymers as well as degrade them (Fig. 1B). Protein quantification was used as a *proxy* for growth because standard monitoring techniques (*e*.*g*. turbidity or colony forming units) could not be performed due to polymer insolubility. Interestingly, *Alcanivorax* sp. 24 was able to grow on all polymers faster than with the labile control substrate, that is, succinate, particularly with PHB (*i*.*e*. ~150 μg of protein/ml at day four; Fig. 1B).

**Table 1:** Characteristics and chemical structure of the polymers used in this study.

Polymer	Form	Formula	Chemical structure	Degradation products	Origin
Poly[3‐hydroxybutyrate] (PHB)^a^	Powder	[C_4_H_6_O_2_]_*n*_	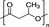	3‐Hydroxybutyrate	Natural
Poly[3‐hydroxybutyrate‐*co*‐3‐hydroxyvalerate] (PHBV)^a^	Pellets	[C_4_H_6_O_2_]_*x*_[C_5_H_8_O_2_]_*y*_	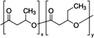	3‐Hydroxybutyrate 3‐Hydroxyvalerate	Natural
Polyethylene succinate (PES)^a^	Chunks	[C_6_H_8_O_4_]_*n*_	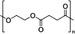	Ethylene glycol Succinate	Synthetic
Polybutylene succinate (PBS)^a^	Pellets	[C_8_H_12_O_4_]_*n*_	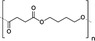	Butanodiol Succinate	Synthetic
Polycaprolactone (PCL)^b^	Chunks	[C_6_H_10_O_2_]_*n*_	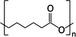	6‐Hydroxyhexanoate	Synthetic
Bis[2‐Hydroxyethyl] terephthalate (BHET)^b^	Flakes	C_12_H_14_O_6_	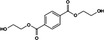	Ethylene glycol Terephthalate (TPA)	Synthetic

a Product supplied by Sigma‐Aldrich®.

b Product supplied by Goodfellow©.

We also assayed if *Alcanivorax* sp. 24 could degrade bis[2‐hydroxyethyl] terephthalate (BHET; Table 1), an intermediate of the recalcitrant polyester polyethylene terephthalate (PET). When grown on BHET, *Alcanivorax* sp. 24 produced apparent clumpy growth in liquid cultures and it decreased the white turbidity of this insoluble compound (Fig. 1C). Unfortunately, BHET interfered with the protein quantification method producing a large background signal, and hence, growth quantification was not possible. To overcome the difficulties in measuring bacterial growth, a metabolic analysis was carried out to determine if the strain was able to degrade the PET intermediate. The hydrolysis of BHET generates ethylene glycol, which could be assimilated by *Alcanivorax*, and terephthalic acid (TPA; Table 1), a sub‐product that should accumulate in the media as the bacterium does not encode for the necessary catabolic pathway for TPA degradation (Zadjelovic *et al*., 2019). BHET and TPA were measured by LC‐MS confirming that *Alcanivorax* sp. 24 was active in hydrolysing the BHET sidechains as ~60% of this compound disappeared from the culture medium and, proportionally, TPA concentration increased (Fig. 1C and Supplementary Fig. S1). The incomplete degradation of BHET and clumping of *Alcanivorax* sp. 24 in the presence of this substrate could be attributed to a possible toxic effect of TPA as it built up in the medium.

### *Proteomic analysis to identify the secreted esterase(s) responsible for polymer hydrolysis*

**Figure 2:**
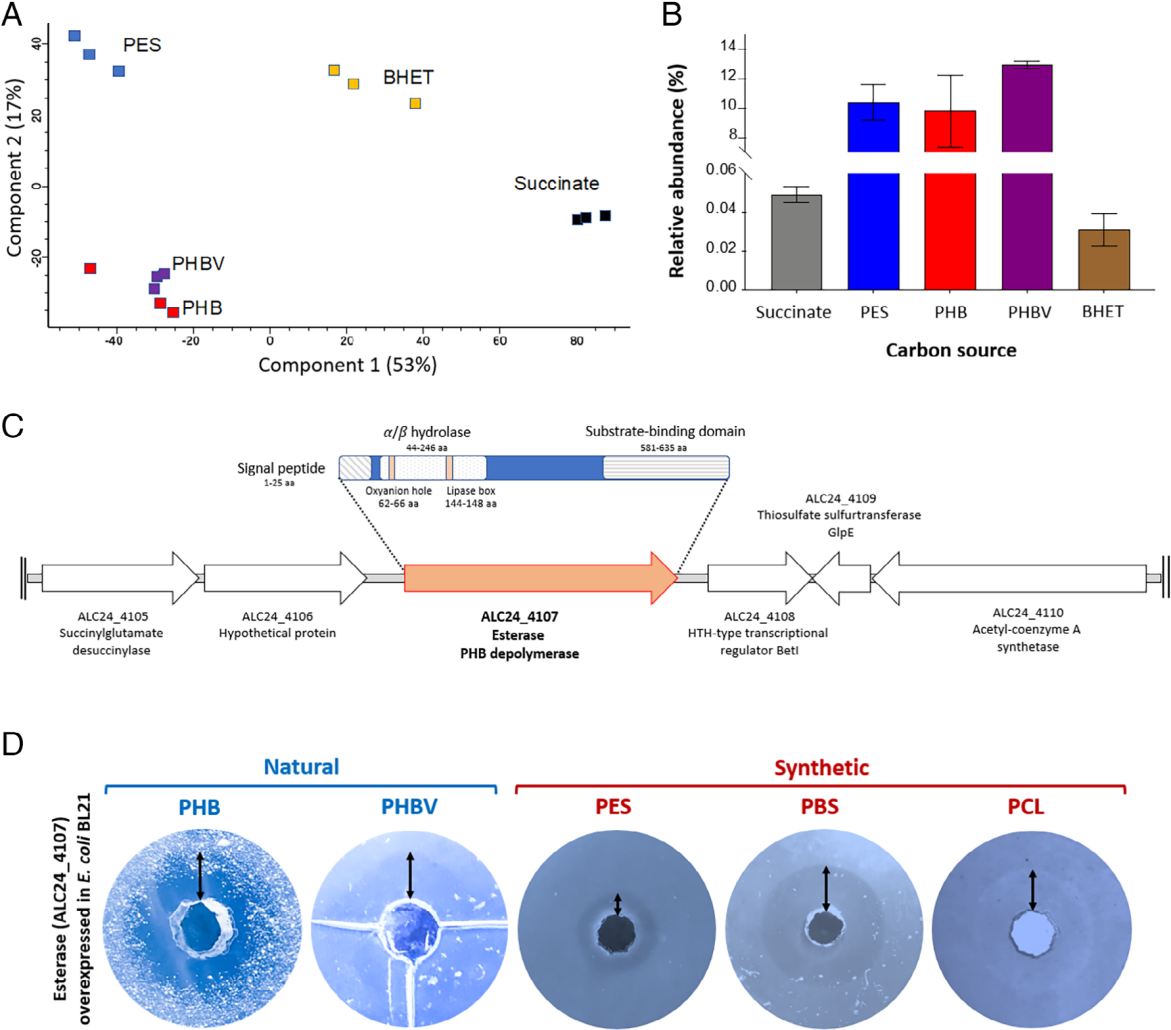
Proteomic analysis and identification of the esterase (*i*.*e*. ALC24_4107) involved in the aliphatic polyester degradation of *Alcanivorax* sp. 24.**A.** PCA of the exoproteomes produced by *Alcanivorax* sp. 24 when grown in the presence of different substrates including the three aliphatic polyesters PES, PHB and PHBV as well as BHET and succinate.**B.** Relative abundance of the esterase ALC24_4107 in each one of the exoproteomes of *Alcanivorax* sp. 24 when grown in the presence of different substrates. Error bars indicate the standard deviation of three biological replicates.**C.** Protein domains and genomic context of ALC24_4107.**D.** Hydrolytic activity of the heterologously overexpressed ALC24_4107 in *E*. *coli* BL21 assessed by a clear zone hydrolysis test on five different aliphatic polyesters. Halos surround 5 mm‐diameter wells. [Color figure can be viewed at http://wileyonlinelibrary.com]

The exoproteomic analysis of *Alcanivorax* grown in the presence of succinate, PHB, PHBV, PES and BHEt allowed the identification of 1250 proteins of which the 13 most abundant ones already represented over 50% of the total exoproteomic fraction, all with a predicted secretion signal (Supplementary Table S1). A PCA of the exoproteomes showed how the conditions PHB, PHBV and PES strongly differed from the labile control (*i*.*e*. succinate) and BHET condition (PC1, which explained 53% of the variability; Fig. 2A). This difference was mainly driven by the abundantly detected PHB depolymerase ALC24_4107 (Fig. 2B). This secreted esterase represented 10%–13% of *Alcanivorax's* exoproteome when grown in the presence of PHB, PHBV and PES as opposed to the other conditions (<0.05% in succinate and BHET), flagging this enzyme as the main candidate in driving the aliphatic polyesters depolymerisation observed in Fig. 1A and, thus, was selected for further investigating (see below). Three other hydrolase/esterase proteins were detected in the exoproteome of *Alcanivorax* sp. 24, although in much lower abundance (*i*.*e*. ALC24_3279, ALC24_3988 and ALC24_4209; Table 2).

**Table 2:** Subset of relevant proteins detected in the exoproteome of *Alcanivorax* sp. 24 when exposed to different polymers.

ID (ALC24_)	Annotated function	Prediction for secretion a			Succinate	PHB			PHBV			PES			BHET		
					Abundance (%; n = 3)	Abundance (%; n = 3)	FC^b^ (log2)		Abundance (%; n = 3)	FC^b^ (log2)		Abundance (%; n = 3)	FC^b^ (log2)		Abundance (%; n = 3)	FC^b^ (log2)	
4107	Esterase PHB depolymerase	SP	NCS	Extracellular	0.05	9.83	6.8	+	12.94	7.4	+	10.40	7.1	+	0.03	−2.0	+
3279	putative hydrolase YxeP	SP	NCS	Unknown	0.01	0.07	0.2		0.07	0.4		0.41	2.8	+	0.15	0.8	
3988	Alpha/beta hydrolase		NCS	Unknown	0.00	0.02	2.7	+	0.04	3.3	+	0.19	5.6	+	0.13	4.0	+
4209	Ferri‐bacillibactin esterase BesA	SP		Unknown	0.00	0.04	3.9	+	0.04	4.5	+	0.01	3.2	+	0.00	−0.7	
2998	Periplasmic binding protein	SP		Unknown	0.32	7.33	4.4	+	8.74	4.7	+	1.42	2.2	+	0.78	0.3	
2082	Alpha‐keto acid periplasmic SBP			Unknown	0.12	1.60	7.6	+	1.60	7.7	+	0.06	0.7		0.00	−3.0	+
2735	C4‐dicarboxylate periplas. SBP	SP		Periplasmic	0.12	0.06	−0.7		0.07	−0.4		3.55	4.7	+	0.10	−1.2	
3132	Alcohol dehydrogenase		NCS	Periplasmic	0.00	0.15	2.7		0.25	3.5	+	13.72	9.3	+	0.94	4.3	+
1432	Alcohol dehydrogenase	SP	NCS	Periplasmic	0.05	0.10	0.0		0.16	0.9		1.38	3.8	+	0.32	0.8	
1781	Flagellin 2		NCS	Extracellular	1.70	10.38	1.7		12.25	2.1	+	10.69	2.0	+	20.46	1.6	
2995	NHL repeat protein	SP	NCS	Outer Memb	0.28	15.79	5.4	+	18.09	5.7	+	2.19	2.3	+	0.41	−0.7	
1102	Type VI secretion system		NCS	Extracellular	0.12	4.84	4.1	+	1.53	3.0	+	0.09	−0.1		1.64	2.7	+
3348	Neisseria PilC protein	SP	NCS	Unknown	0.03	0.55	2.8	+	1.39	4.2	+	0.55	3.1	+	0.10	−0.1	
2260	Fimbria adhesin protein	SP	NCS	Extracellular	0.08	1.04	2.8	+	0.60	2.2	+	0.16	0.6		0.30	0.3	
0797	Unknown, adherence	SP	NCS	Extracellular	0.00	0.16	4.9	+	0.19	5.2	+	0.21	5.4	+	0.16	4.3	+
2342	hypothetical protein			Unknown	0.00	0.71	11.3	+	3.26	13.6	+	0.39	10.4	+	0.00	1.0	

a Protein secretion systems and localization. SP, signal peptide. NCS, non‐classical secretion.

b Fold change (FC) of each protein in each one of the conditions *vs*. the succinate control. Significant values are indicated (+; *q*‐value < 0.05).

The exoproteomic analysis also highlighted the substrate‐binding component of three membrane transporters that could be involved in importing the specific subproducts from polymer hydrolysis (Table 2). Although transporters ALC24_2998 and, more specifically, ALC24_2082 could be involved in transporting 3‐hydroxybutyrate and 3‐hydroxyvalerate from PHB and PHBV depolymerisation, ALC24_2735 may import derivatives generated from PES hydrolysis. The strong increase of alcohol dehydrogenases in the periplasm of the bacterium in the presence of PES (ALC24_3132 and ALC24_1432 representing 13.7% and 1.4% protein abundance respectively; Table 2) suggests that its degradation product ethylene glycol is transformed into glyoxylate before being imported and catabolized within the cell (Salvador *et al*., 2019). Interestingly, the alcohol dehydrogenase ALC24_3132 was also significantly upregulated when *Alcanivorax* was grown in the presence of BHET (log2 fold change of 4.3), suggesting a possible generation of ethylene glycol from the hydrolysis of BHET as hinted by the detection of TPA during the metabolomics analysis (Fig. 1C). An increased secretion of proteins involved in adhesion when *Alcanivorax* sp. 24 was grown in the presence of the different aliphatic polyesters was also detected (*e*.*g*. ALC24_3348, ALC24_2260 and ALC24_0797; Table 2). Although the proteomic data has helped us identify those enzymes and transporters that are possibly involved in assimilating polyester intermediates, further biochemical experimentation is required to confirm their function and substrate specificity.

A previously characterized esterase from *Alcanivorax borkumensis* that showed a strong hydrolytic activity on aliphatic polyesters (ABO2449; Hajighasemi *et al*., 2016) and that had a conserved homologue in our *Alcanivorax* sp. 24, *i*.*e*. ALC_2069 (*E*‐value 10^−118^), was not detected in the exoproteome under any condition (Supplementary Table S1). We analysed the cellular proteome of *Alcanivorax* sp. 24 (Supplementary Table S2) to investigate if the enzyme ALC_2069, which we initially believed was responsible for the observed polyester hydrolysis, was contained within the cell. The cellular proteome produced 2590 detected proteins amongst which ALC_2069, although detected, only represented <0.004% of the proteome.

The proteomic data highlighted ALC24_4107 as the main candidate responsible for the phenotype observed in Fig. 1A, and hence, this enzyme was selected for further confirmation and characterization.

### *Protein structure and genomic context of the abundantly secreted esterase ALC24_4107*

Apart from a clear signal peptide for secretion on the protein's N‐terminal (25 amino acids‐long signal peptide involved in conventional secretion systems), ALC24_4107 contained an α/β‐hydrolase domain between amino acids leucine and threonine (position 44 to 246; Fig. 2C). Although α/β‐hydrolase domains are a diverse superfamily which includes esterases, proteases, lipases, dehalogenases and epoxide‐hydrolases, ALC24_4107 contained the characteristic catalytic triad of amino acids serine, aspartic acid and histidine found in the active site of hydrolases involved in polyester degradation (Jendrossek, 1998). The enzyme's structural analysis also revealed the presence of a substrate‐binding domain in position 581 to 635 (Fig. 2C) which is essential for the hydrolysis of non‐soluble substrates – such as when the polyesters are found in the extracellular milieu (Shinomiya *et al*., 1997) – and may provide the enzyme with its substrate specificity.

ALC24_4107 was analysed using the Depolymerase Engineering Database, DED (Knoll *et al*., 2009), revealing it belonged to the extracellular depolymerase group e‐dPHAscl (type 1) homologous family 8. Nevertheless, ALC24_4107 and its closest homologues (*i*.*e*. AAB40611.1 from *Alcaligenes faecalis* and *Alcanivorax dieselolei*) formed a distinctive branch within the family of depolymerases (Supplementary Fig. S2).

The genomic context of the esterase ALC24_4107 in *Alcanivorax* sp. 24 showed a transcriptional regulator immediately downstream from the gene (Fig. 2C) which could be involved in regulating the strong induction of the enzyme observed by proteomics. Enzymes involved in short‐chain fatty acid metabolism, and which may have a role in processing the hydrolysed intermediates of the polyesters, were also encoded further downstream from ALC24_4107.

### *ALC24_4107 has a promiscuous hydrolytic activity on aliphatic polyesters*

To confirm the hydrolytic activity of esterase ALC24_4107, the enzyme was overexpressed in *E*. *coli* BL21. Although the enzyme was successfully produced and secreted when it was cloned with its original signal peptide for secretion, best results were obtained when this peptide was replaced by the host's *pho*A signal peptide (Supplementary Fig. S3; Ahn *et al*., 2006). The highest overexpression, assessed by clear zone halos produced by *E*. *coli* supernatants on PHB agarose plates, were obtained after 72 h of incubation after inducing with 1 mM of IPTG at 27°C (Supplementary Fig. S3).

The overexpressed esterase degraded all polymers *i*.*e*. PHB, PHBV, PES, PBS and PCL (Fig. 2D) confirming the broad range activity of this enzyme on aliphatic polyesters.

### *Distribution and activity of homologous esterases of ALC24_4107 in other bacteria*

**Figure 3:**
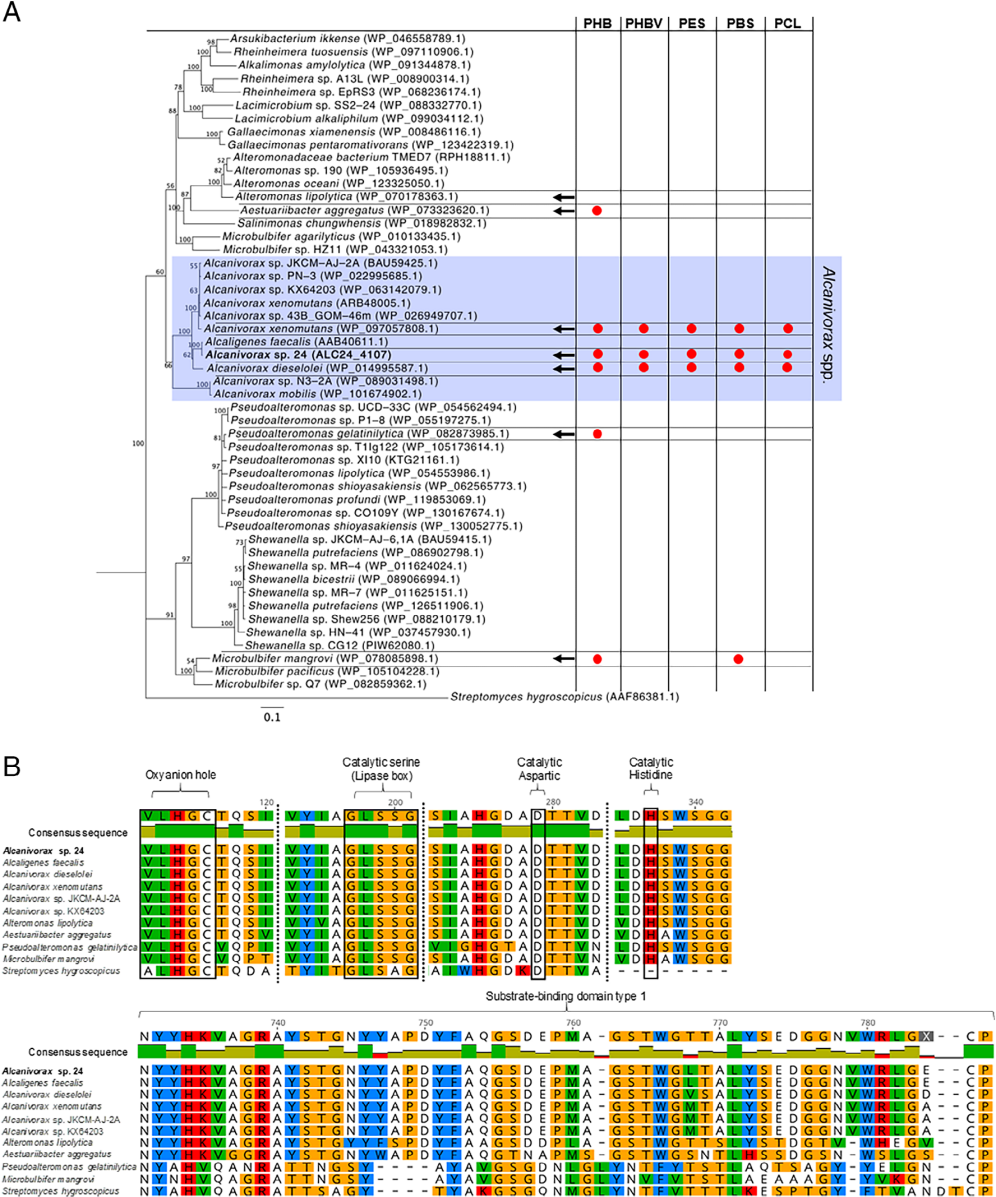
Similarities and hydrolytic activity of ALC24_4107 homologous esterases.**A.** Phylogenetic tree of ALC24_4107 and its closest homologues in other bacteria retrieved from an NCBI BLASTp search. The tree was generated using Neighbour‐Joining and Jukes‐Cantor as the generic distance, with bootstrap set to 1000 replicates represented at the base of the nodes, and using the PHB‐depolymerase from *Streptomyces hygrogroscopicus* as the outgroup. Arrows indicate the strains that were purchased and for which hydrolytic activity was assessed on each one of the five aliphatic polyesters. The tests that gave a positive clearing halo (information available in Supplementary Fig. S4) are indicated by red circles.**B.** Multiple alignment of ALC24_4107 with 10 relevant homologues, including the esterases encoded by the six strains tested for their hydrolytic activity. Only the catalytic and substrate‐binding domains are shown. [Color figure can be viewed at http://wileyonlinelibrary.com]

A BLASTp search of the esterase ALC24_4107 showed the presence of homologous copies of the enzyme in other *Alcanivorax* strains (*i*.*e*. with 71% to 93% identity), as well as in other genus, that is, *Pseudoalteromonas*, *Aestuariibacter*, *Microbulbifer* and *Alteromonas* (55%, 58%, 59% and 60% identity respectively; Fig. 3A). However, the closest related protein of ALC24_4107 found in the NCBI databases was a PHB depolymerase encoded by *Alcaligenes faecalis* (99% identity). All proteins included in the phylogenetic tree were annotated as PHB depolymerases or had esterase‐like domains (Fig. 3A). Interestingly, no homologue of ALC24_4107 was observed in the model strain *Alcanivorax borkumensis*.

Six strains encoding esterases from different branches of the phylogenetic tree (Fig. 3A) were purchased in an effort to determine their ability to degrade the different aliphatic polyesters. Out of the six strains assessed, only the *Alcanivorax* isolates (*Alcanivorax xenomutans* JC109 and *Alcanivorax dieselolei* B‐5) were able to produce clear zone halos in all polymers, that is, PHB, PHBV, PES, PBS and PCL (Fig. 3A and Supplementary Fig. S4). Those strains encoding esterases from different branches (*i*.*e*. *Microbulbifer mangrovi* DD13, *Pseudoalteromonas gelatinilytica* NH153 and *Aestuariibacter aggregatus* WH169) only produced clearing halos on PHB plates, although *M*. *mangrovi* also produced halos on PBS (Supplementary Fig. S4). Despite the absence of halos could be due to a lack of enzyme induction, it is most likely that the homologous PHB depolymerases from different branches are not as promiscuous as those encoded by *Alcanivorax*. The sequence analysis of each one of the tested esterases revealed that, although they all conserved the catalytic triad of amino acids, the substrate binding domain diverged between the copies encoded by *Alcanivorax* and the rest of the strains (Fig. 3B).

## Discussion

Over the past few decades, the identification of PHA depolymerases has received large attention (Knoll *et al*., 2009; García‐Hidalgo *et al*., 2013; Martínez‐Tobón *et al*., 2018; Sayyed *et al*., 2019). Despite the extant sequence variability amongst these enzymes, all intra‐ and extracellular PHA depolymerases share a common α/β‐hydrolase fold and a catalytic triad (Knoll *et al*., 2009). The key finding of this study is the vast promiscuity of substrates shown by the abundantly secreted PHA depolymerase ALC24_4107, which is conserved in a number of *Alcanivorax* strains (Fig. 3). This enzyme, highly induced in the presence of polymers (representing over >10% of the exoproteome; Fig. 2B), was able to hydrolyse a large range of aliphatic polyesters of natural and synthetic origin, that is, PHB, PHBV, PES, PBS and PCL (as proven by heterologous overexpression; Fig. 2D). It had already been reported that *Alcanivorax* strains could degrade a variety of these polyesters (*i*.*e*. PHB, PBS and PCL) (Sekiguchi *et al*., 2011), although the mechanisms involved were not previously identified. In another study that performed an *in vitro* screening for PLA esterases, researchers identified that the enzyme ABO2449 encoded by *Alcanivorax borkumensis* had a strong hydrolytic activity on PLA as well as on a range of other aliphatic polyesters, that is, PHBV, PCL and PES (Hajighasemi *et al*., 2016). A homologue of the ABO2449 α/β‐hydrolase was encoded by *Alcanivorax* sp. 24 (ALC_2069) although this enzyme was not detected in the exoproteome (Supplementary Table S1), and its abundance was extremely low in the cellular proteome (< 0.004%; Supplementary Table S2). Hence, we prove here that ALC24_4107, and not ALC_2069, is responsible for aliphatic polyester biodegradation by *Alcanivorax* sp. 24.

Biosynthesis of natural polyesters, that is, PHAs, is a widely distributed mechanism of carbon storage amongst environmental microorganisms (Jendrossek and Pfeiffer, 2014), and therefore, it may be an important source of carbon and energy to those organisms able to biodegrade them when PHA producers are lysed, for example, after phage infection or inefficient grazing. For example, 95% of members of the Roseobacter clade, an abundant and versatile group of marine heterotrophs (Buchan *et al*., 2005; Christie‐Oleza *et al*., 2012) that pioneer the colonization of marine surfaces including marine plastic debris (Elifantz *et al*., 2013; Erni‐Cassola *et al*., 2019), encode *pha*C, the polymerase that catalyses PHA biosynthesis (*i*.*e*. 711 of 750 Roseobacter genomes encoded a *pha*C homologue, *E*‐value <10^−100^; Supplementary Table S3). *Alcanivorax* sp. 24 also encodes *pha*C (ALC24_0403 and ALC24_1241) as well as for the other genes involved in PHA biosynthesis (*i*.*e*. *pha*A, *pha*B and phasin) and, hence, is likely to produce and store PHAs as proven in other *Alcanivorax* strains (Fernández‐Martínez *et al*., 2003; Sabirova *et al*., 2006). *Alcanivorax* sp. 24, isolated from marine plastic debris, may be able to persist and thrive in marine biofilms by assimilating natural PHAs produced by its surrounding microorganisms as well as biodegrade the material it colonizes if made of polyester chains.

**Figure 4:**
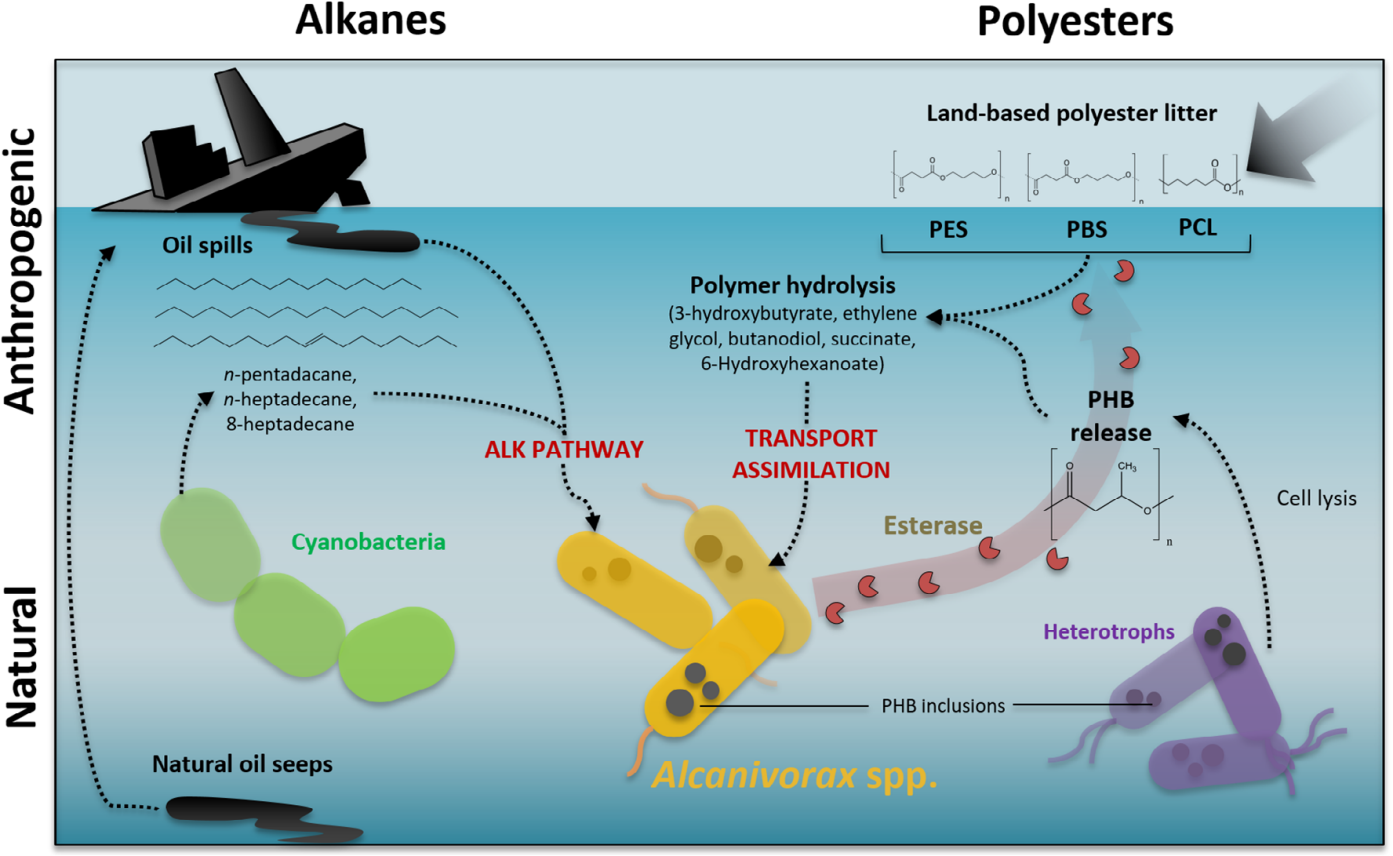
Ecological strategy of *Alcanivorax* spp. in the environment.The biodegradation and assimilation of hydrocarbons (left) and polyesters (right) of both natural (bottom) and anthropogenic origin (top) is depicted. [Color figure can be viewed at http://wileyonlinelibrary.com]

Because its isolation and characterization, the genus *Alcanivorax* has become a reference of marine oil‐degradation (Yakimov *et al*., 1998; de Lorenzo, 2006; Schneiker *et al*., 2006; Gregson *et al*., 2019). Although *Alcanivorax* spp. are found in low abundance under normal environmental conditions, these microbes rapidly bloom in oil‐contaminated marine ecosystems (Kasai *et al*., 2002; Hara *et al*., 2003). Although *Alcanivorax* shows a clear preference for oils and has been classified as an ‘obligate’ hydrocarbonoclastic organism (Yakimov *et al*., 2007), members of this genus can also grow on some more‐labile substrates (Radwan *et al*., 2019), although they are likely to be outcompeted by other members of the marine microbial community. It was suggested that *Alcanivorax* may be able to persist in pristine environments by using alkanes produced by marine cyanobacteria (Lea‐Smith *et al*., 2015; Valentine and Reddy, 2015). Nevertheless, in this study, we show that, as well as an oil biodegrader, *Alcanivorax* is a specialist in biodegrading aliphatic polyesters via the secretion of an abundant and substrate‐promiscuous α/β‐hydrolase (Fig. 4). Here, we suggest that this esterase would allow this organism to persist using naturally occurring polyesters and, currently, it may also confer the potential to biodegrade polyester plastics of anthropogenic origin.

## Experimental procedures

### *Bacterial strains and culture conditions*

*Alcanivorax* sp. 24 (accession number SNUA0000000; Zadjelovic *et al*., 2019) as well as the six strains obtained from the Korean and German culture collections KCTC and DSMZ [*i*.*e*. *Microbulbifer mangrovi* DD13 (KCTC 23483), *Alcanivorax xenomutans* JC109 (KCTC 23751), *Alteromonas lipolytica* JW12 (KCTC 52408), *Alcanivorax dieselolei* B‐5 (DSM 16502), *Aestuariibacter aggregatus* WH169 (DSM 23094), *Pseudoalteromonas gelatinilytica* NH153 (DSM 100951)] were routinely grown in Marine Broth or on Marine Agar plates (BD Difco™). *E*. *coli* BL21 was grown using LB broth (Sigma‐Aldrich®).

The analysis of polymer degradation was performed with Bushnell‐Hass basal mineral media (BH; Bushnell and Haas, 1941), adjusted to pH 7.0 and supplemented with NaCl 30 g L^−1^ and 1 ml L^−1^ of an ASW trace metal solution (Wyman *et al*., 1985). Agarose 1% (w/v) was added as the solidifying agent. BH mineral media was amended with sodium succinate 0.5% w/v or each one of the polymers listed in Table 1 at 0.3% w/v as the source of carbon and energy. BHET was added at a final concentration of 0.1% w/v.

### *Polymer preparation and biodegradation test (clear zone test)*

Aliphatic polyesters were purchased from Sigma‐Aldrich® and Goodfellow©. Polymers were available in the forms detailed in Table 1 and emulsified in the mineral media as previously described (Nishida *et al*., 1998; Tansengco and Tokiwa, 1998). Briefly, 0.3% w/v of PHB was directly added as a powder suspension to BH media containing 1% agarose (Nishida and Tokiwa, 1993), autoclaved and mechanically homogenized by blending before pouring into Petri dishes. PBS, PES and PCL were pre‐dissolved in acetone and PHBV in dichloromethane before adding at 0.3% final polymer concentration to autoclaved media, after which it was blended and poured into Petri dishes. Dissolvent was evaporated for 20 min at 80°C.

Clear zone degradation tests were conducted as described previously (Augusta *et al*., 1993). Briefly, wells (approximately 5 mm diameter) were made in the solid media in which resuspended cells in minimal BH medium were used as the inoculum. Inoculated plates were left to dry and then incubated at 30°C for 7 days.

### *Growth curves and monitoring*

The liquid cultures were prepared in 50 ml glass Erlenmeyer flasks filled with 30 ml of BH mineral media containing the different sources of carbon and inoculated with washed bacterial cells, all performed in independent biological triplicates. Cultures were incubated for 7 days at 30°C and shaking at 200 rpm. Due to the clumps formed by the insoluble polymers, growth was monitored by protein quantification using the QuantiPro™ BCA Assay kit (Sigma‐Aldrich®) following the supplier's recommendations. LIVE/DEAD Cell Viability Assay (Invitrogen™) was used to visualize polymer colonization and check the cell viability (Supplementary Fig. S5). A *T*‐test analysis was performed using Prism 7 to determine the significance of the growth curve results (*p*‐value set at *p* ≤ 0.05).

Degradation of BHET was performed as described for other polymers although the monitoring of growth was not possible by protein quantification due to the large background signal given by the substrate. BHET degradation was measured via metabolite consumption and TPA formation using LC‐MS as described below.

### *Metabolite detection of BHET and TPA using LC‐MS*

Samples were prepared and processed as previously described (Yoshida *et al*., 2016) with minor modifications. BHET and TPA were extracted from 5 ml of culture samples using 10 ml of ethyl acetate as a solvent. The organic phase was recovered and evaporated (speedVac, Genevac™ EZ‐2). The residual product was resuspended in a mix of 150 μl of dimethyl sulfoxide (DMSO), 100 μl of 16 mM phosphate buffer adjusted at pH 2.5 and 50 μl of acetonitrile (total resuspension volume 300 μl). The sample was acidified using HCl (pH 2.0) and pre‐filtered (0.22 μm Spin Cups‐Cellulose Acetate Filters) before analysis via reversed‐phase liquid chromatography on a Dionex UltiMate 3000 HPLC (ThermoScientific) equipped with a Zorbax Eclipse Plus C18 column (dimensions 4.6 mm × 150 mm, 5 μm particle size; Agilent Technologies) coupled to an amaZon SL Ion Trap MS (Bruker), operated in positive mode with a scanning range for molecular ions of 100–1000 *m*/*z*. A gradient elution of mobile phases A (water, 0.1% formic acid) and B (acetonitrile, 0.1% formic acid) was used at a flow rate of 1 ml min^−1^ as follows: the ratio of solvent A:B was lineally decreased from 95:5 to 30:70 over 5 min, followed by a second decrease from 30:70 to 20:80 over 10 min and finally from 20:80 to 5:95 over 12 min. The injection volume was 10 μl at a temperature of 25°C. MS data was processed with the Bruker Compass DataAnalysis software version 4.2 (Bruker). Calibration curves were constructed using different concentrations of BHET and TPA in DMSO (Supplementary Fig. S1).

### *Exoproteome and cellular proteome preparation for proteomics*

At day 7, 20 ml of the culture from each culture was centrifuged at 4000 rpm, 15 min at 4°C. Cell pellets were immediately frozen on dry ice and stored at −20°C. Supernatants (exoproteomes) were further filtered through 0.22 μm pore size hydrophilic filters (Minisart® Syringe Filters). Proteins in the supernatants were precipitated using a trichloroacetic acid (TCA) and sodium deoxycholate (DOC) protocol as previously described (Christie‐Oleza and Armengaud, 2010). Both exoproteome and cell pellets were dissolved in 70 μl and 300 μl of lithium dodecyl sulphate (1 × LDS with 1% β‐mercaptoethanol, Invitrogen™) respectively. Samples were incubated at 95°C for 5 min and vortexed for three cycles before loading 30 μl of each sample onto 10% NuPAGE™ Bis‐Tris precast gels (1.0 mm, 10‐well, Invitrogen™). Gels were allowed a short migration to enter the polyacrylamide gel (1 cm) before staining with SimplyBlue™ SafeStain (Invitrogen™). Band sections containing the entire proteome were cut and placed into 1.5 ml microcentrifuge tubes to be stored at −20°C until use (Christie‐Oleza and Armengaud, 2010).

### *Tryptic digestion and shotgun proteomics*

Proteomes contained in the gel bands were reduced (dithiothreitol) and alkylated (iodoacetamide) before digestion using trypsin (Christie‐Oleza and Armengaud, 2010). Tryptic peptides were recovered from the gels using an extraction buffer (formic acid/acetonitrile; (Shevchenko *et al*., 2007) and analysed by nanoLC‐ESI‐MS/MS using an Ultimate 2000 LC system (Dionex‐LC Packings) coupled to an Orbitrap Fusion mass spectrometer (Thermo‐Scientific). An LC separation of 60 min for exoproteomes or 120 min for cellular proteomes were performed on a 25 cm column before MS/MS analysis using settings as described previously (Christie‐Oleza *et al*., 2015).

### *Comparative proteomic analysis*

Peptide spectrum profiles were identified and quantified using MaxQuant (v1.5.5.1) within the framework of the Label Free Quantification (LFQ) method (Cox and Mann, 2008). Parameters were set by default although including the match between run function. Spectra were searched against the protein database of *Alcanivorax* sp. 24 (Zadjelovic *et al*., 2019). Perseus (v1.5.6.0) was used to perform the comparative proteomic analysis (Tyanova *et al*., 2016), where a two‐sample *T*‐test was used to determine protein variations between each condition when compared to the succinate control condition. Statistical analysis was set using a false discovery rate (FDR) of 0.05 and minimal log2 fold change of 2. A protein was considered valid when present in every replicate of at least one condition. The list of polypeptides, LFQ intensities, relative abundance and differential detection is listed in Supplementary Tables S1 and S2.

### *Heterologous overexpression of ALC24_4107 in *E*. *coli* BL21*

The esterase gene ALC24_4107 was codon optimized, and three variants were synthesized by GenScript: (1) using the wild type signal‐peptide for secretion, (2) using no signal‐peptide, and (3) using a signal‐peptide for secretion from the host's *pho*A (MKQSTIALALLPLLFTPVTKA; Ahn *et al*., 2006). Synthesized genes were ligated into the NdeI/XhoI restriction sites of the overexpression vector pET‐24a(+). Overexpression of ALC24_4107 in *E*. *coli* BL21 was optimized by testing different temperatures (27 and 37°C) and IPTG concentrations (0.2, 0.5 and 1 mM). IPTG induction was carried out when cultures reached an OD_600_ of 0.6. The culture cell pellet and supernatant of each one of the conditions were recovered after 24 h by centrifugation (8000 rpm, 5 min). Cell pellets were disrupted using 1× BugBuster® Protein Extraction Reagent in combination with three sonication steps of 5 min. Supernatants and cell lysates were screened for their hydrolytic activity using the clear zone test on PHB plates (Supplementary Fig. S3). After determining the optimal conditions for ALC24_4107 overexpression (*i*.*e*. supernatants from cultures containing the *pho*A signal peptide, incubated at 37°C and induced with 1 mM of IPTG), its activity was tested against polyesters PHB, PHBV, PES, PBS and PCL using the clear zone test (Fig. 2D).

### *Proteomic and genomic in silico analysis*

The motifs and domains in ALC24_4107 were searched using the Conserved Domain Analysis tool from NCBI. Protein secretion was predicted using the servers: SignalP 4.1. (Petersen *et al*., 2011), SecretomeP 2.0a (Bendtsen *et al*., 2005), LipoP 1.0. (Juncker *et al*., 2003) and PSORTb v3.0.2. (Yu *et al*., 2010). The phylogenetic tree of ALC24_4107 and its closest homologues in the NCBI database (as determined by BLASTp) was built using Geneious Prime applying Jukes‐Cantor and Neighbour‐Joining.

## Supporting information


**Fig S1** BHET degradation by *Alcanivorax* sp. 24 assessed by LC–MS. (**A**) BHET standard curve. (**B**) Extracted ion chromatogram of BHET and TPA obtained from cultures where *Alcanivorax* sp. 24 was absent (panels 1 and 2) and present (panels 3 and 4).
**Fig. S2.** Phylogenetic context of the PHB‐depolymerase ALC24_4101 from *Alcanivorax* sp. 24 and *Alcanivorax dieselolei* (highlighted in blue) with the closest PHA depolymerases identified by the Depolymerase Engineering Database (DED; Knoll *et al*., 2009). The sequence AAB40611.1 from *Alcaligenes faecalis* and homologues from *Shewanella* sp. MR4 and MR7 (ABI40356.1 and ABI41661.1, respectively) –all three present in the DED database– are also highlighted in blue. The tree was generated using Neighbour‐Joining and Jukes‐Cantor as the generic distance, with bootstrap set to 1000 replicates represented at the base of the nodes. The scale bar shows nucleotide/amino acids substitutions per 100 residues.
**Fig. S3.** PHB clear zone test to screen for the activity of the heterologously overexpressed esterase from *Alcanivorax* sp. 24 (ALC24_4107) in *E*. *coli* BL21. Plastic square petri dishes 13 × 13 cm.
**Fig. S4.** Aliphatic polyester clear zone test using microorganisms encoding close homologue esterases to ALC24_4107. Polymers PHB (A), PESu (B), PBSu (C), PHBV (D) and PCL (E) were tested. *Alcanivorax* sp. 24 on PHBV and PCL (F and G, respectively) were performed separately.
**Fig. S5.** Determination of cells viability using LIVE/DEAD™ *Bac*Light™ Bacterial Viability Kit. Staining procedure applied to *Alcanivorax* sp. 24 biofilms grown on PHB, PES and BHET. Green staining represents viable cells whereas red staining represents dead cell or those with compromised membrane integrity.

**Supplementary Table S1** Exoproteome of *Alcanivorax* sp. 24 exposed to different polyesters.

**Supplementary Table S2** Cellular proteome of *Alcanivorax* sp. 24 exposed to different polyesters.

**Supplementary Table S3** Presence of *pha*C as a proxy of PHA biosynthesis in 750 *Roseobacter* genomes.
