# Supplementary material for: Beyond oil degradation: enzymatic potential of Alcanivorax to degrade natural and synthetic polyesters
Source: Environ Microbiol. 2020 Feb 27;22(4):1356–69. doi: 10.1111/1462-2920.14947 (PMC7187450; doi:10.1111/1462-2920.14947)
Supplement: Supplementary file 1 — Fig S1 BHET degradation by Alcanivorax sp. 24 assessed by LC–MS. (A) BHET standard curve. (B) Extracted ion chromatogram of BHET and TPA obtained from cultures where Alcanivorax sp. 24 was absent (panels 1 and 2) and present (panels 3 and 4). Fig. S2. Phylogenetic context of the PHB‐depolymerase ALC24_4101 from Alcanivorax sp. 24 and Alcanivorax dieselolei (highlighted in blue) with the closest PHA depolymerases identified by the Depolymerase Engineering Database (DED; Knoll et al., 2009). The sequence AAB40611.1 from Alcaligenes faecalis and homologues from Shewanella sp. MR4 and MR7 (ABI40356.1 and ABI41661.1, respectively) –all three present in the DED database– are also highlighted in blue. The tree was generated using Neighbour‐Joining and Jukes‐Cantor as the generic distance, with bootstrap set to 1000 replicates represented at the base of the nodes. The scale bar shows nucleotide/amino acids substitutions per 100 residues. Fig. S3. PHB clear zone test to screen for the activity of the heterologously overexpressed esterase from Alcanivorax sp. 24 (ALC24_4107) in E. coli BL21. Plastic square petri dishes 13 × 13 cm. Fig. S4. Aliphatic polyester clear zone test using microorganisms encoding close homologue esterases to ALC24_4107. Polymers PHB (A), PESu (B), PBSu (C), PHBV (D) and PCL (E) were tested. Alcanivorax sp. 24 on PHBV and PCL (F and G, respectively) were performed separately. Fig. S5. Determination of cells viability using LIVE/DEAD™ BacLight™ Bacterial Viability Kit. Staining procedure applied to Alcanivorax sp. 24 biofilms grown on PHB, PES and BHET. Green staining represents viable cells whereas red staining represents dead cell or those with compromised membrane integrity. [file EMI-22-1356-s001.pdf]

Supplementary information

Beyond oil degradation: Enzymatic potential of *Alcanivorax* sp. 24 to degrade other natural and synthetic polymers.

Vinko Zadjelovic*[1], Audam Chhun[1], Mussa Quareshy[1], Eleonora Silvano[1], Juan R. Hernandez-Fernaud[1,2], María M. Aguilo-Ferretjans[1,3], Rafael Bosch[3,4], Cristina Dorador[5,6,7], Matthew I. Gibson[8,9] and Joseph A. Christie-Oleza*[1,3,4]

[1] School of Life Sciences, University of Warwick, U.K
[2] Unidad de investigación-HUC, La Laguna-Tenerife, Spain
[3] Department of Biology, University of the Balearic Islands, Spain
[4] IMEDEA (CSIC-UIB), Esporles, Spain
[5] Laboratorio de Complejidad Microbiana y Ecología Funcional, Universidad de Antofagasta, Chile
[6] Departamento de Biotecnología, Universidad de Antofagasta, Chile
[7] Centre for Biotechnology & Bioengineering (CeBiB), Santiago, Chile
[8] Department of Chemistry, University of Warwick, U.K.
[9] Warwick Medical School, University of Warwick, U.K.

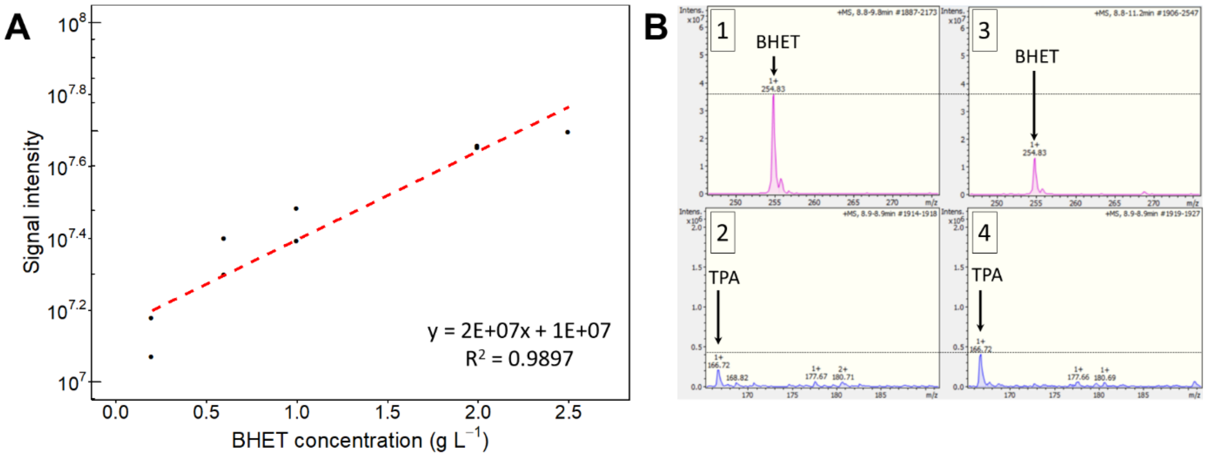

Fig S1. BHET degradation by *Alcanivorax* sp. 24 assessed by LC-MS. (**A**) BHET standard curve. (**B**) Extracted ion chromatogram of BHET and TPA obtained from cultures where *Alcanivorax* sp. 24 was absent (panels 1 and 2) and present (panels 3 and 4).

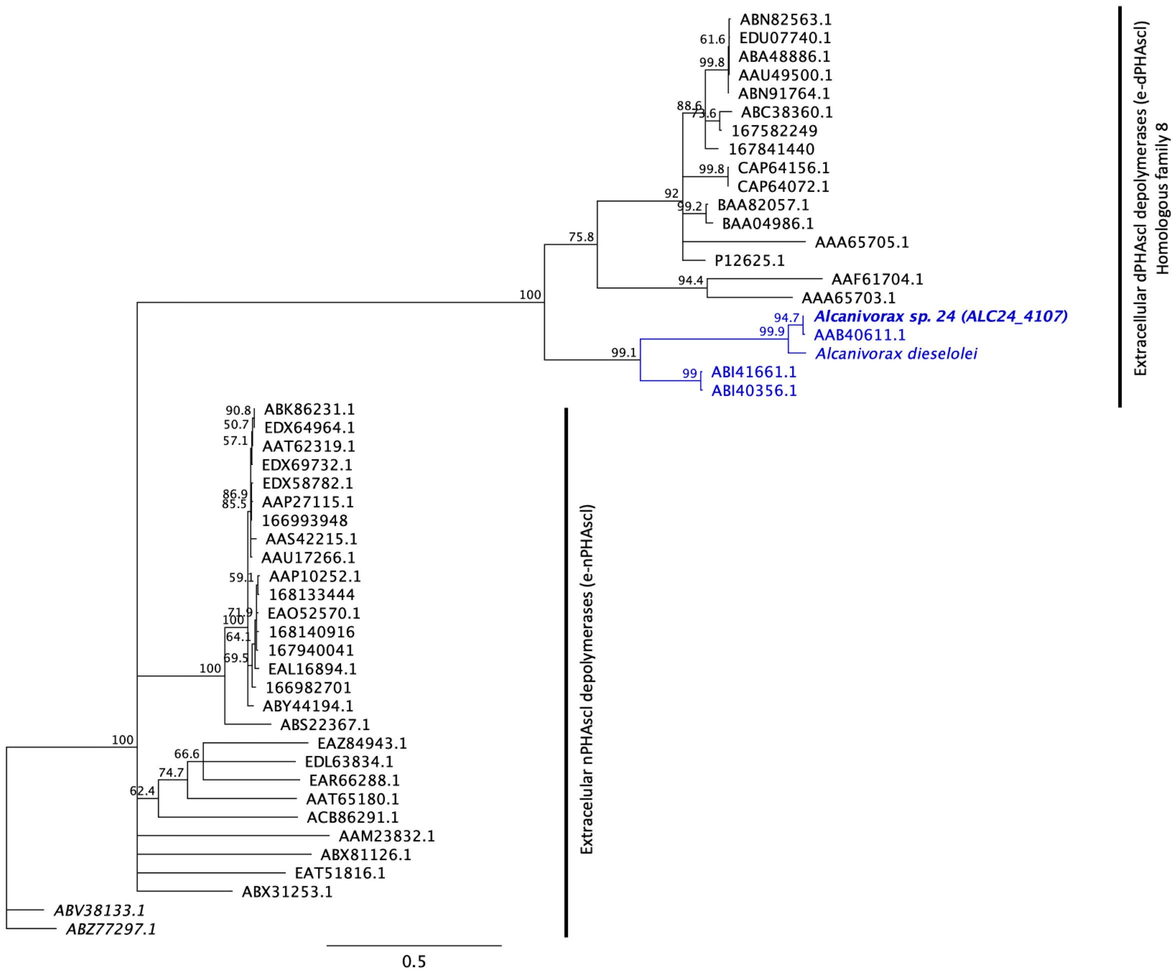

Fig. S2. Phylogenetic context of the PHB-depolymerase ALC24_4101 from *Alcanivorax* sp. 24 and *Alcanivorax dieselolei* (highlighted in blue) with the closest PHA depolymerases identified by the Depolymerase Engineering Database (DED; Knoll et al., 2009). The sequence AAB40611.1 from *Alcaligenes faecalis* and homologues from *Shewanella* sp. MR4 and MR7 (ABI40356.1 and ABI41661.1, respectively) –all three present in the DED database– are also highlighted in blue. The tree was generated using Neighbour-Joining and Jukes-Cantor as the generic distance, with bootstrap set to 1000 replicates represented at the base of the nodes. The scale bar shows nucleotide/amino acids substitutions per 100 residues.

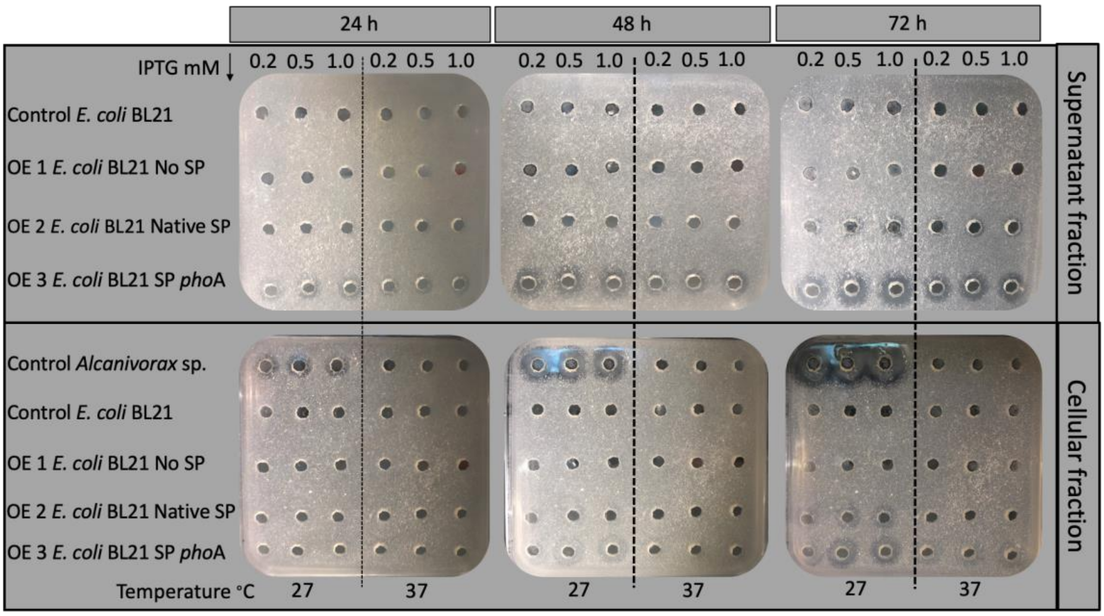

Fig. S3. PHB clear zone test to screen for the activity of the heterologously overexpressed esterase from *Alcanivorax* sp. 24 (ALC24_4107) in *E. coli* BL21. Plastic square petri dishes 13×13 cm.

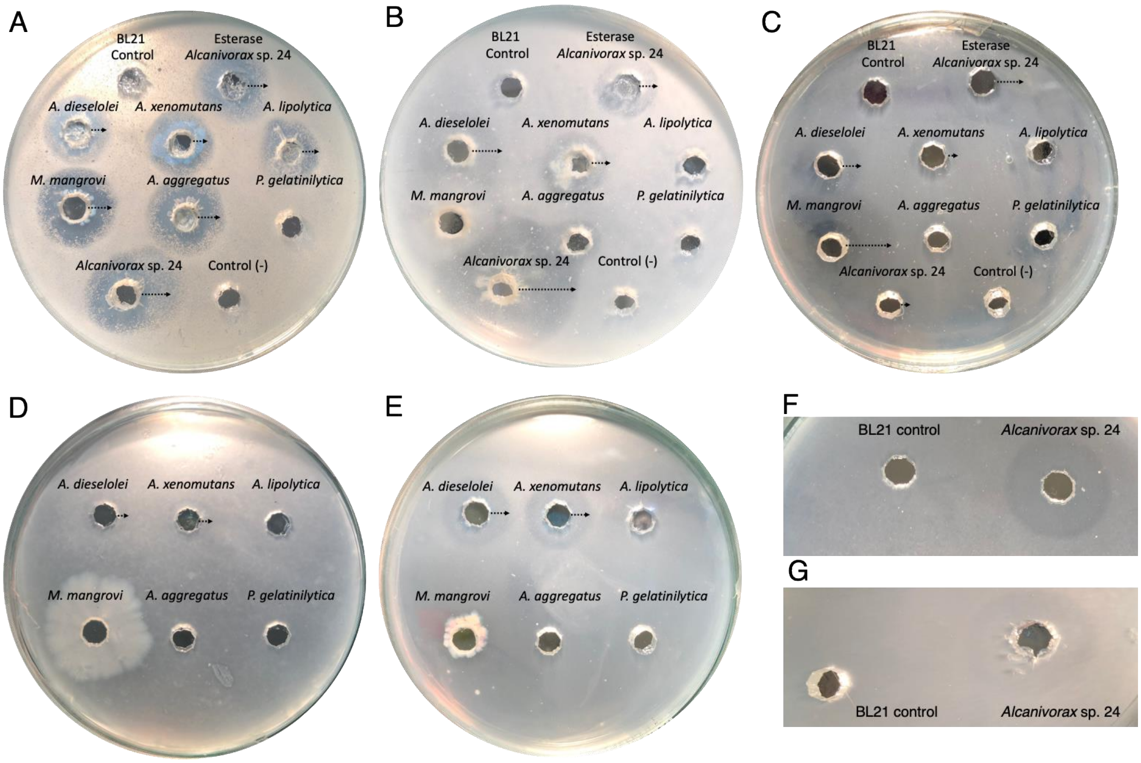

Fig. S4. Aliphatic polyester clear zone test using microorganisms encoding close homologue esterases to ALC24_4107. Polymers PHB (A), PESu (B), PBSu (C), PHBV (D) and PCL (E) were tested. *Alcanivorax* sp. 24 on PHBV and PCL (F and G, respectively) were performed separately.

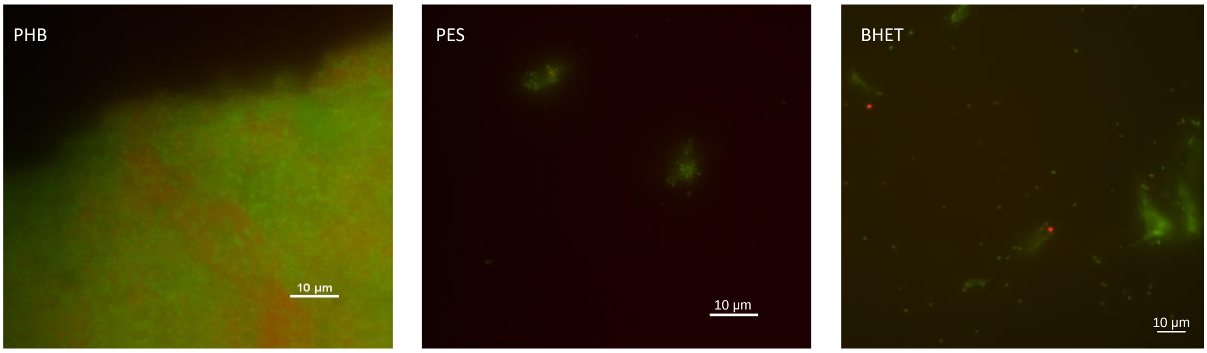

Fig. S5. Determination of cells viability using LIVE/DEAD™ *Bac*Light™ Bacterial Viability Kit. Staining procedure applied to *Alcanivorax* sp. 24 biofilms grown on PHB, PES and BHET. Green staining represents viable cells whereas red staining represents dead cell or those with compromised membrane integrity.
